# Multivariate Analysis on Physical Activity, Emotional and Health Status of University Students Caused by COVID-19 Confinement

**DOI:** 10.3390/ijerph191711016

**Published:** 2022-09-03

**Authors:** Bethy Merchán-Sanmartín, Mayra Brocel-Bajaña, Johny Pambabay-Calero, Sergio Bauz-Olvera, Néstor Montalván-Burbano, Maribel Aguilar-Aguilar, Paúl Carrión-Mero

**Affiliations:** 1 Centro de Investigaciones y Proyectos Aplicados a las Ciencias de la Tierra (CIPAT), ESPOL Polytechnic University, Guayaquil 09015863, Ecuador; 2 Facultad de Ingeniería en Ciencias de la Tierra (FICT), ESPOL Polytechnic University, Guayaquil 9015863, Ecuador; 3 Geo-Recursos y Aplicaciones (GIGA), ESPOL Polytechnic University, Guayaquil 09015863, Ecuador; 4 Facultad de Ciencias Naturales y Matemáticas (FCNM), ESPOL Polytechnic University, Guayaquil 09015863, Ecuador; 5 Department of Economy and Business, University of Almería, Ctra. Sacramento s/n, La Cañada de San Urbano, 04120 Almería, Spain; 6 Innovation, Management, Marketing and Knowledge Economy Research I2Maker, ESPOL Polytechnic University, Guayaquil 9015863, Ecuador

**Keywords:** pandemic, physical exercise, sedentarism, anxiety, depression, undergraduates

## Abstract

Confinement as a result of COVID-19 had a strong impact around the world and restricted mobility. The university community started to take routine classes in a virtual and sedentary way, causing negative effects on their health and habits. The objective of this research is to analyze the impact of confinement through surveys of students and interviews with university professors, in order to study the effects of confinement on physical activity, emotional state, and health. The methodology was as follows: (i) preliminary data; (ii) survey development, interviews, and information collection; (iii) data processing and multivariate presentation of the results, using multiple correspondence analysis (MCA) and multiple factor analysis (MFA). The results of 375 respondents show that there is a low level of physical activity (<300 METs, 49.6%), where women register sedentary behavior (73%). Emotionally, most of them show feeling bored. Some express anxiety, depression, discomfort, and frustration. In terms of health, there are rheumatic, circulatory, respiratory, and other diseases related to obesity. It is essential to create programs that promote physical exercise to reduce the consequences of sedentary lifestyles on the physical, social, and mental health of university students, especially engineering students, who experienced greater effects of confinement than those studying nutrition and social sciences.

## 1. Introduction

Human beings have a series of capacities (cognitive, functional, motor, emotional, and psychosocial) that allow them to adapt to the environment and respond to its demands. Cognitive abilities allow us to learn and remember information, and to organize and solve problems [1]. The functional capacity allows us to perform the basic and instrumental activities of daily living [2,3]. Motor capacities permit us to perform movements in a coordinated manner [4]. Emotional intelligence (EI) consents us to perceive, use, understand, and regulate emotions [5]. Finally, in the biopsychosocial aspect, the human being has a complex mentality based on various aspects, such as values, conscience, ethics, motivations, desires, and personality. These are characteristics that allow an individual to be in a social organization (family, friends, community, nation, and social groups) and to behave according to his psychological interests and the limits presented by his body and his learning [6].

Physical activity (PA) has a beneficial effect on brain development and cognitive function [7]. Activity has been researched in the last 30 years to help humans improve in different aspects of life. Cognitive improvements and brain health are essential to provide quality of life, educational and professional opportunities, and the ability to make decisions in any area of life [8,9,10]. According to the European Council [11], sport is defined as any physical activity that, through participation, organized or otherwise, is aimed at the expression or improvement of physical and mental fitness, the development of social relations, or the achievement of results in competitions at all levels. The four most important types of PA for health are those shown in Figure S1 (see Supplementary Materials), which include daytime activities, such as climbing stairs, performing tasks at home or work, walking or cycling, and recreational activities [12,13,14].

Cardiovascular work, shown in Figure S1 (see Supplementary Materials), can stimulate the heart to increase the amount of oxygenated blood sent to working muscles and cells [15]. Aerobic activities, such as swimming, jogging, brisk walking, and cycling, improve oxygen transport to the body’s cells [16].

One study concluded that physical activity has the potential to target tumor growth through the regulation of immune and inflammatory functions, and that exercise can be pursued as a treatment for cancer [17]. Furthermore, intervention studies show that physical training is associated with numerous positive outcomes, as follows: loss of body weight, improved fitness levels, better sleep quality, reduced anxiety and depression, and an increased sense of patient empowerment [18,19]. In short, physical activity is an excellent stimulus for the proper functioning of all human capacities.

However, in December 2019, in Wuhan, China, the first cases of an infectious disease of unknown etiology, characterized by pneumonia and caused by severe acute respiratory syndrome (SARS), commonly recognized by the name of Coronavirus 2019 (COVID 2019) were identified [20]. Since then, it has affected dozens of countries and cities worldwide. The Johns Hopkins Coronavirus Resource Center (CRC) indicates that the 12 most-affected countries were the US, France, India, Brazil, Germany, Italy, Russia, the United Kingdom, Spain, Turkey, the Netherlands, and Japan [21]. As of 16 August 2022, the number of COVID 2019 cases has reached 588,757,628 cases, and there have been 6,433,794 cumulative deaths worldwide [22].

The first infection in Latin America occurred in Brazil on 25 February 2020 [23]. The government authorities took the necessary measures to prevent the spread of the virus, but they were not fast enough, as contagion was imminent. Furthermore, these measures prevented people’s free access to education, work, mobility, and social life.

This lifestyle has contributed to reducing or decreasing physical activity levels and increasing sedentary lifestyles, mainly in work and study [24]. Excessive use of electronic equipment, such as computers, cell phones, video game consoles, and televisions promotes stationary behaviour [25]. In a study conducted in Canada [26], children and youth played less outdoors, participated in more screen-based recreational activities, and slept more during the initial COVID-19 outbreak than before the restrictions. Physical inactivity could be a significant cause of disease, as studies show prolonged sitting is associated with an increased risk of heart disease, obesity, diabetes, cancer, and even premature death [27,28,29,30,31,32]. Sedentary behaviour has become the world’s fourth leading cause of death [33].

During the COVID-19 pandemic, several areas of human life have been affected. A survey study conducted on Facebook in 2020 indicated that a group of 1970 participants from Taiwan experienced sleep disturbances, which worsened their physical health and caused them to exhibit suicidal thoughts [34]. Athletes in the Wielkopolska region reported that running at night facilitates the reconciliation of sleep and improves sleep quality [35]. In Spain, several studies, one of them in adults over 65, indicated that a sedentary lifestyle worsens the physical health status of people with diabetes and obesity [36]. Another study, carried out on 4195 participants between 15 and 35 years, found a higher risk of developing anxiety, depression, and other mental disorders in inactive young people [37]. Likewise, another study indicates that 72% of 4180 Spaniards presented psychological discomfort, with those who work outside the home showing more significant discomfort than those who usually stay home [38]. The results of a study of 3052 U.S. adults presented symptoms of depression and anxiety [39]. In Peru, 21.7% healthcare workers experienced severe anxiety, whereas 26.1% of them experienced severe mental distress [40].

Several universities have studied students’ personal experiences on how the CO-VID-19 pandemic has affected their lives. One example is the study of university students in China, which shows that confinement has had significant effects on psychological well-being and high anxiety levels [41].

On the other hand, stressors influencing university students in Malaysia include financial constraints, remote online teaching, and uncertainty related to their academic performance and future career prospects [42]. In 14 universities in Turkey, the highest percentage of inactive students reported high stress, mild generalized anxiety, and low life satisfaction [43]. Students at the University of Extremadura-UNEX, Spain, show academic dissatisfaction, as they point out that some professors have overloaded them with subjects and others have not provided them with guidance on how their subjects will be taught [44]. In another university in Spain, 75% of 78 students reduced their physical activity, manifesting a decrease in their mood and physical and mental energy. In Peru, university students presented symptoms, such as tiredness, fatigue, sleep disorders (nightmares or insomnia), lack of motivation to undertake academic tasks, headache, and pessimism about the future [45].

University teachers must change their teaching habits to provide a better service that ensures students’ physical, academic, and psychological well-being. The Group of Online Teaching Managers of the Public Universities of Castilla y León has prepared a guide of recommendations to help teachers and universities in this process [46]. In addition, the University of Almeria in Spain offered a series of keys to work on resilience to improve the psychological and academic environment of students [47].

In Ecuador, according to the Servicio Nacional de Gestión de Riesgos y Emergencias (SNGRE), as of 31 July 2022, there have been 972,832 confirmed cases and 35,810 deaths [48]. The country went through a collapse in the health system and a change in the lifestyle of the inhabitants, where, as is common in most countries, a massive quarantine measure was implemented to prevent the spread of the virus. However, despite reducing the number of infections, the population has faced conditions of emotional disorders, depression, stress, low mood, irritability, insomnia, and post-traumatic stress [49]. At the academic level, as of March 2020, classes for all levels were suspended in the country, generating a drastic transition from the face-to-face modality to the virtual modality [50].

Specifically, it is still necessary to work on guaranteeing the psychological and academic environment of students and teachers at all levels. With the present study, it will be possible to detect which factors have been the most affected due to confinement in university students, to determine what characteristics the students most affected by COVID-19 have, and what role physical activity play in the state of the person.

The objective of this research is to analyze the impact of COVID-19 confinement through the elaboration and application of surveys to students, as well as interviews with university professors, to study the effects on the physical activity and emotional and health status of a university campus.

**Figure 1:**
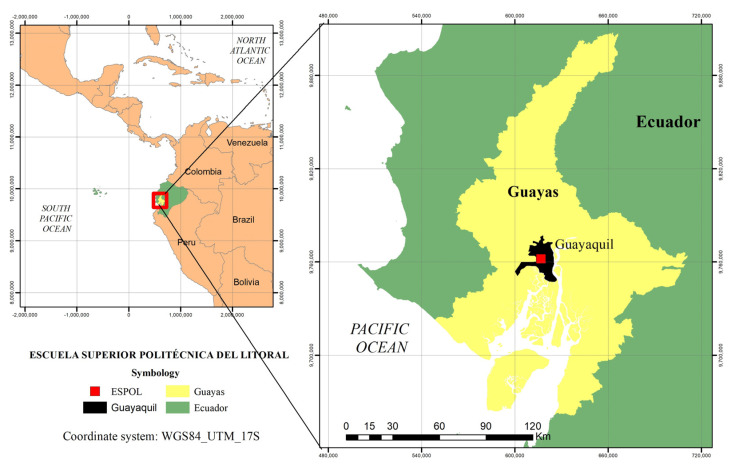
Geographical location of the ESPOL university campus.

The case study was conducted at the ESPOL Polytechnic University located in the northeast of the city of Guayaquil, Guayas, Ecuador, at the Gustavo Galindo Velasco Campus (Km. 30.5 of the perimeter road), as shown in Figure 1. According to the QS Latin America University Rankings 2021 [51], ESPOL is ranked No. 74 among 411 universities in Latin America [52]. In addition, the Green Metric 2021 [53] ranks ESPOL as the No. 1 university in Ecuador in terms of sustainability. Like other universities, ESPOL has given importance to physical activities from its beginnings to the present, providing scholarships to athletes and including in its curriculum complementary training subjects.

There are currently eight faculties (the acronyms correspond to the name of the faculty in Spanish and are identified in this way by the university community), as follows: the Faculty of Art, Design, and Audio-visual Communication (FADCOM), the Faculty of Life Sciences (FCV), the Faculty of Natural Sciences and Mathematics (FCNM), the Faculty of Social and Humanistic Sciences (FCSH), the Faculty of Earth Sciences Engineering (FICT), the Faculty of Electrical and Computer Engineering (FIEC), the Faculty of Mechanical Engineering and Production Sciences (FIMCP), the Faculty of Maritime Engineering and Marine Sciences (FIMCM).

## 2. Material and Methods

**Figure 2:**
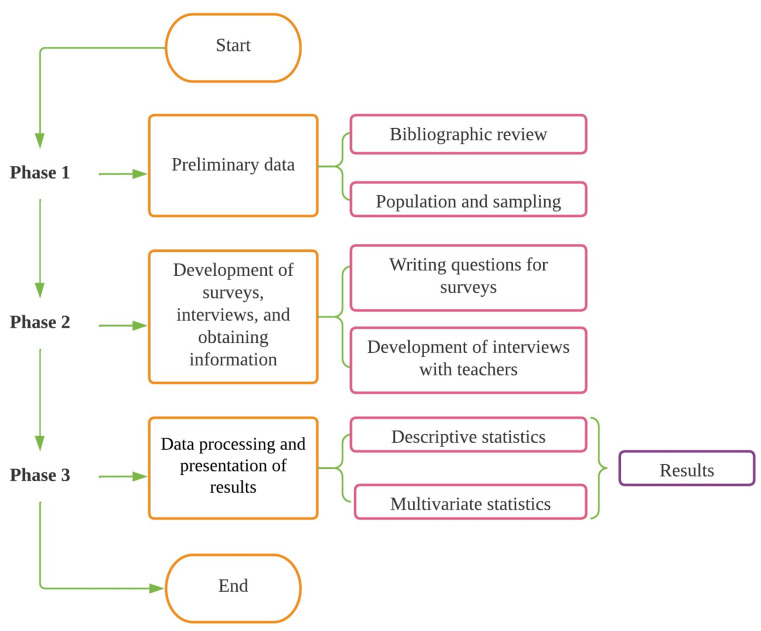
Data science diagram.

This work contemplates a methodological process consisting of three phases (see Figure 2), as follows: (i) preliminary data, in terms of the scope of the study, population, and sample size; (ii) survey development, interviews, and information gathering; (iii) data processing and multivariate presentation of results.

### 2.1. Preliminary Data: Scope of the Study, Population, and Sample Size

This research assessed the physical activity, emotional, and health impacts on young students during COVID-19, presenting the results of a large-scale, rapid survey using multivariate statistical plots.

For this purpose, an exploratory qualitative and quantitative study using probabilistic sampling was carried out, with the universe being the eight faculties. The study period was from 25 June to 11 October 2021. First, the universe of the population is the total number of students. Second, the authors determined the sample size, the confidence level, and the sampling error. Formulas are in Table S1 in the Supplementary Material. Finally, using simple random sampling, the students agreed to take the survey freely and voluntarily.

### 2.2. Surveys, Interviews and Data Collection

#### 2.2.1. Survey Development

At this stage, we used a survey with a previously validated questionnaire [54,55,56] aimed at undergraduate students at the Escuela Superior Politécnica del Litoral. The survey is in Spanish because this is the language in which classes are taught to Ecuadorian students and to the few international students whose native language is Spanish. The authors sent the survey online through institutional mail using the Google Forms application. In order to reach the sample number immediately and so that no exogenous variables affected the responses, the research was carried out in the months of low academic load, and a reminder was sent via email each month. We excluded students who did not complete the entire questionnaire.

#### 2.2.2. Elaboration of Survey Questions

Details of the survey questions are in Table S2 in the Supplementary Materials. The form has 18 semi-structured questions with the following guidelines:

- Five aspects are classified as follows: socio-demographic, health, emotional, sporting, plus a personal question.
- From the socio-demographic aspect, the following information was requested: gender, age, marital status, nationality, enrolment number, and information on whether or not they work.
- Through a medical history from Sworn Junior College, we determine the illnesses they suffer or have suffered [54]; questions taken from the Madrid Health COVID-19 survey were also asked [55] to find out if living habits have changed due to the confinement.
- For the emotional aspect, they were asked about their experience at home; questions based on the Madrid Health COVID-19 survey were used [55].
- The sports aspect was measured using the abbreviated version of the International Physical Activity Questionnaire (IPAQ) [56]. There, they were asked about the types of activities done before the pandemic and those done during the pandemic; additionally, they were asked the days per week and minutes per day that they perform vigorous and moderate physical activity. With this, we obtained the metabolic equivalent of task (MET) according to the answers given by the participant. For example, activity at 3.3 METs is considered walking, 4 METs is considered moderate physical activity, while 8 METs is considered vigorous physical activity. Respondents were classified into three groups: those with a low level of physical activity, of less than 300 MET/week, those with moderate physical activity, of more than 300 MET and less than 1500 MET/week, and those with a high level of physical activity of more than 1500 MET/week. The hours spent sitting during the day were also queried to assess sedentary (more than 6 h) or non-sedentary (less than 6 h) behavior.

A.F. Low = 3.3 METs × minutes of walking × days per week A.F. Moderate = 4 METs × minutes × days per week A.F. Vigorous = 8 METs × minutes × days per week Total = A.F. Low + A.F. Moderate + A.F. Vigorous(1)

**Figure 3:**
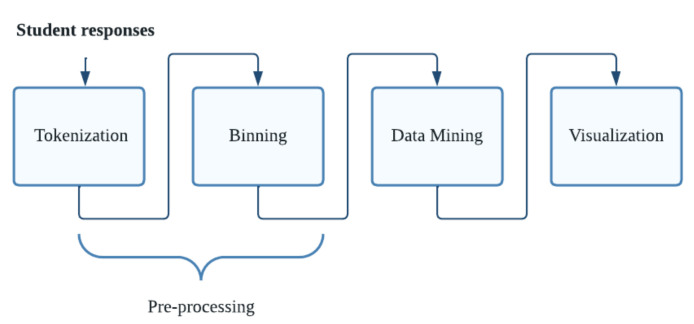
Process for obtaining a word cloud.

In the personal free-response question, we applied a text mining process to the open-ended question (the aspect of their life that they felt most affected by the confinement), where we obtained the word frequency of the answers given by the students in a word cloud graph. In Figure 3, we show the process. The preprocessing task consists of separating the words without connectors, punctuation, stop words, XML tags, lowercase, and special characters (tokenization), and creating the body of refined text (binning) to be able to carry out a data mining process. Finally, the visualization is carried out through a word cloud chart that shows a set of ideas or concepts according to their importance. Less essential words have more discreet sizes and often go unnoticed. For their part, the most important words have larger sizes and are able to better capture attention. Once the word cloud was obtained, the most representative terms were chosen [57].

#### 2.2.3. Survey Validation

The co-authors, which include teachers, researchers, and students, conducted a survey analysis beforehand to ensure that the survey fitted the objective of the article and took no more than 5 min to complete.

We conducted an exploratory analysis using a factor analysis technique for dimension reduction. In this sense, the value of the Kaiser–Meyer–Olkin (KMO) measure of sampling adequacy statistic is 0.71. The Cronbach’s alpha value represents the internal consistency of the test, i.e., the degree to which the test items covary with each other was not estimated because we used a questionnaire previously validated by [58], where the value was 0.97 and 0.98 for both sexes. As a result, the correlation coefficient of the items with the Pearson biplot dimensions was between 0.50 and 0.81, giving the instrument a strong reproducibility capacity. In the same sense, the cumulative percentage of variance for the first two dimensions was around 16%. Table 1 shows the eigenvalues and percentage of accumulated variance per dimension. 

#### 2.2.4. Teacher Interviews

Teachers who served during the pandemic asked five free-ranging questions. Based on the questions asked of the students, we wanted to explore the recommendations given by teachers of complementary subjects related to physical activity. Questions 1 and 4 are to discover the teacher’s expertise and how many years the university has been offering sporting activities. Question 5 is to find out what difficulties they have experienced when teaching physical exercise virtually. Finally, questions 2, 3, and 6 are for recommendations to students on how to combat sedentary lifestyles (See Table S3 in Supplementary Materials).

### 2.3. Data Processing and Multivariate Presentation of Results

**Figure 4:**
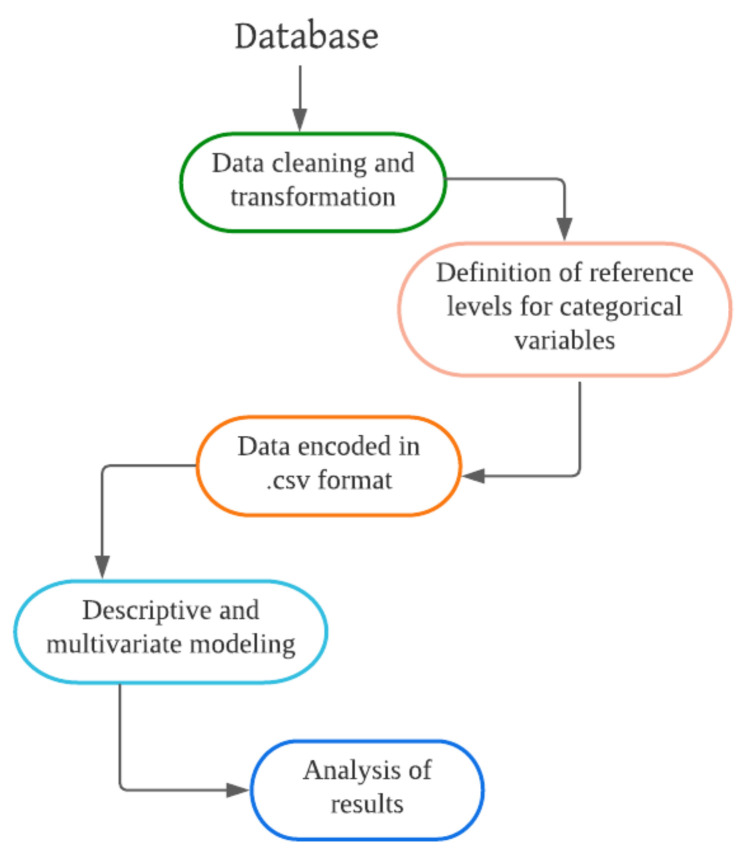
Data processing and multivariate presentation.

The data required for this study were cleaned and transformed. In addition, the multiple-choice categorical variables established reference levels were stored in a .csv file. Finally, we used descriptive and multivariate statistics for the analysis of the results (see Figure 4). We obtained a percentage distribution of the 18 semi-structured questions from the descriptive analysis. For the multivariate analysis, we used the multiple factorial analysis (MFA) model and the multiple correspondence analysis (MCA). We were able to compare the results obtained in each of them.

Multiple factor analysis (MFA) studies several groups of variables defined on the same set of individuals. The core of the method is a factor analysis applied to the whole set of variables, in which each group of variables is weighted. This point of view leads to a representation of individuals and variables, as in any factor analysis. In addition, this induces a visualization with representations of the set of individuals associated with each group of variables superimposed [59,60].

Here, ACM allows the determination of the combinations of variables and modalities most frequently represented in the analysis. The MCA is used to analyze a set of observations described by a group of nonlinear nominal variables. Each nominal variable comprises several modalities, each of which is coded as a binary variable [61].

## 3. Results

The total population was 10,433 students. The confidence level of 95% with a 5% error, the probability of occurrence and non-occurrence of the event studied was 0.5, and the Z-value was 1.96. Therefore, the sample size when applying the population adjustment was 371 persons. We sent surveys to 1321 people, of which 376 responded, and 375 were included in this studio.

### 3.1. Socio-Demographic Data

Of the 375 students, the majority were women who were not working. The ages ranged from 16 to 38 years, where more than 95% were single and of Ecuadorian nationality. The details of these results, by age, can be seen in Table 2.

The main results will be presented below using multivariate statistical analysis.

### 3.2. Multivariate Analysis of Factors Associated with the Pandemic: Diseases, Lifestyle Habits, Emotional State, and Physical Activity

#### 3.2.1. Relationship between Physical Activity and Disease Progression during the Pandemic

**Figure 5:**
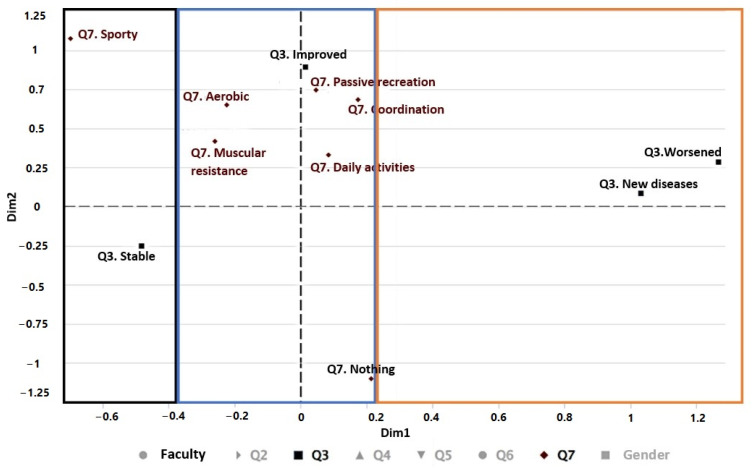
Multiple correspondence analysis (MCA) of the variables of faculty, Q3, and Q7.

Figure 5 shows the relationship between the physical activity (Q7) performed during the pandemic with the evolution of the student’s diseases (Q3). It can be seen in the first gradient (black color) that, during the confinement, the students who remained to do sports are in stable health. On the other hand, in the second gradient (blue color) those who have engaged in aerobic exercise (jogging, walking, or cycling), passive recreation (board games or playing instruments), daily activities (housework), coordination (dancing), or muscular endurance (lifting weights) have improved their health. On the contrary, in the third gradient (orange color), it can seem that those who have not done any activity have worsened their health or have had new diseases.

#### 3.2.2. Relationship between Lifestyle Habits and Emotional State

**Figure 6:**
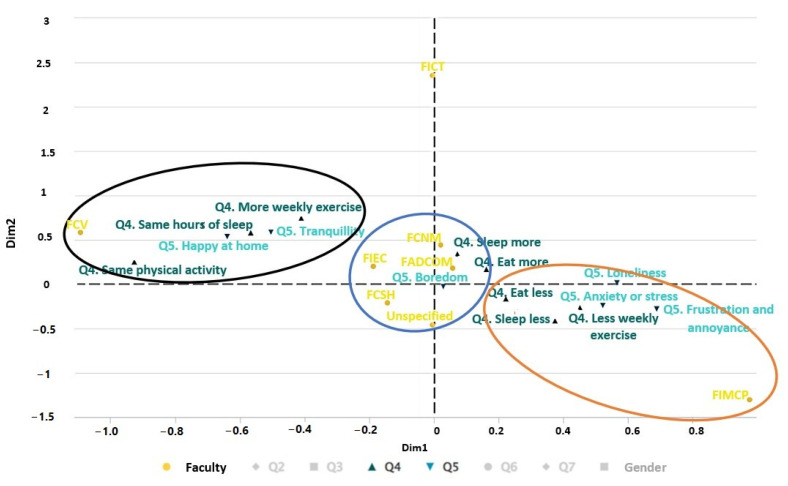
Multiple correspondence analysis (MCA) of the variables of faculty, Q4, and Q5.

Figure 6 shows the relationship by faculty between the change in habits during confinement (Q4) and the student’s mood (Q5). In the first cluster (black color), it can be seen that FCV students feel happy to be at home and do more weekly exercise, and their hours of rest and physical activity remain the same before and after the pandemic. In the second cluster (blue color), most students from the FCNM, FADCOM, FIEC, and FCSH faculties report feeling bored, sleeping more, and eating more during the pandemic. Finally, in the third cluster (orange color), although there were few respondents from the FIMCP faculty, they responded that they feel loneliness, anxiety, stress, frustration, and discomfort. In addition, during the pandemic, they eat less and sleep less than before.

#### 3.2.3. Pre-Existing Diseases and their Evolution during Confinement

**Figure 7:**
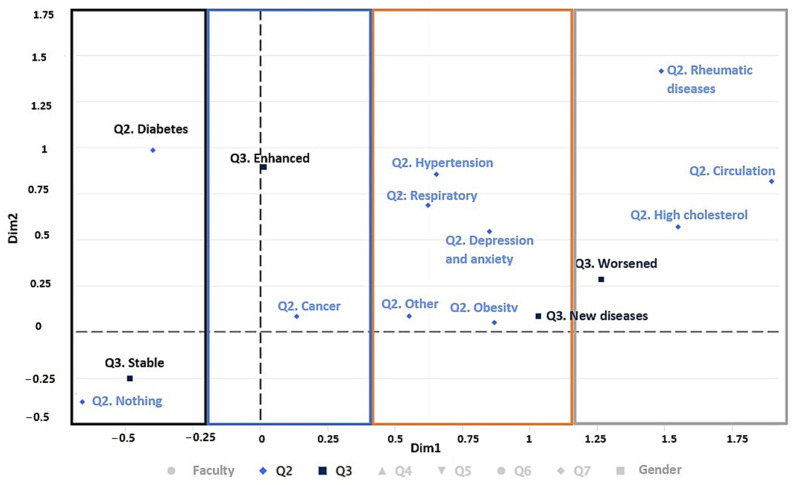
Multiple correspondence analysis (MCA) of the variables of faculty, Q2, and Q3.

Figure 7 shows how COVID-19 confinement has affected students with diseases (Q2) and their evolution during confinement (Q3). In the first gradient, on the left side (black color), it can be seen how people who have had diabetes or no disease are stable during the period of confinement. In the second gradient (blue color), those who have had cancer improved during the pandemic. In the third gradient (orange color), which represents those who have had hypertension, obesity, respiratory diseases, depression, anxiety, and other illnesses, new illnesses have appeared during the pandemic. Finally, in the fourth gradient (grey color), circulatory problems and high cholesterol have worsened during the pandemic for those who have had rheumatic diseases.

#### 3.2.4. Variation in Pre-Existing Diseases, Emotional state, and Living Habits during Confinement

**Figure 8:**
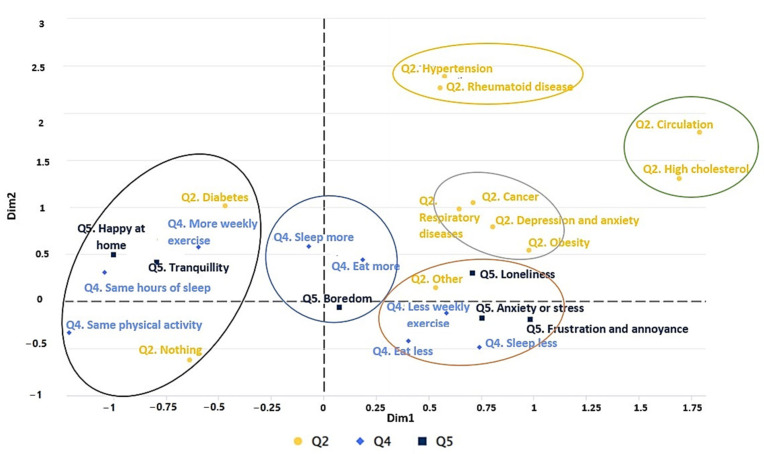
Multiple factor analysis (MFA) of the variables Q2, Q4, and Q5.

Figure 8 shows the emotional state (Q5) and living habits (Q4) of students with diseases (Q2) during confinement. In the first cluster (black color), one can see that the students with diabetes and no disease, who are calm at home, do more weekly exercise and have slept as before during confinement. In the second cluster (blue color), students reported boredom, more sleeping than before, and more eating than before, during confinement. In the third cluster (orange color), students who reported loneliness, frustration, discomfort, anxiety, and stress exercised less weekly, ate less than before, and slept less than before. In the fourth cluster (grey color), a high association between diseases can be seen, as those who have cancer also suffer from depression, anxiety, and obesity. Finally, in the fifth cluster (yellow color), those with hypertension suffer from rheumatic diseases.

#### 3.2.5. Variation in Physical Activity before and during the Pandemic

**Figure 9:**
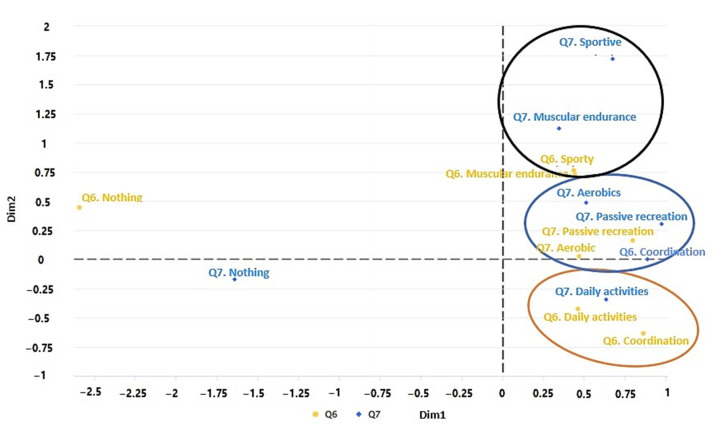
Multiple factor analysis (MFA) of the variables of faculty, Q6, Q7.

Figure 9 shows the variation of physical activity performed before (Q6) and during (Q7) the pandemic. In the first cluster (black color), one can see that the students who did sports and muscular endurance before the pandemic mostly kept doing only muscular endurance exercises during the pandemic. In the second cluster (blue), students who were doing aerobic exercise and passive recreation before the pandemic have continued with passive recreation and aerobic and coordination exercises during the pandemic. Finally, in the third cluster (orange color), students who engaged in daily activities and coordination exercises before the pandemic now only maintain daily activities during the pandemic.

To test the stochastic independence of the different categories in the study, the χ^2^ (chi-square) statistic was used to determine if the null hypothesis of the contrast related to the independence of categories should be rejected, which is synthesized as follows:

**H_0_:**  *Category A is stochastically independent of category B vs. H_1_*.

**H_1_:**  *Categories A and B are not stochastically independent*.

Table 3, which shows the results of the tests of the stochastic independence hypothesis and contrasts the χ^2^ (chi-square) with their respective *p*-values, is shown below.

### 3.3. Sports Aspect: Levels of Physical Activity and Sedentary Lifestyle

Table 4 shows that most women have low levels of physical activity, and most men have moderate levels of physical activity. Among men and women, men have a high level of physical activity compared to women. Regarding behaviour, we found that most of the 375 respondents have a sedentary lifestyle. Women had more passive behaviour than men.

As expected, COVID confinement significantly decreased physical activity, mainly because it restricted free mobility and reduced activities, such as foraging for food and medicine. Men drastically decreased their attendance in sports activities (from 45% to 15%) and had to start helping with household chores (11% to 20%). However, there was no significant change in the increase in daily activities but, instead, data revealed that they did no physical activity at all (from 8% to 22%) compared to women (from 7% to 16%). These values correspond with the results of sedentary behaviour, which revealed that about 73% of females have sedentary behaviour, as they spend more than 6 h a day on academic activities (Table 5).

From an emotional point of view, the data show significant differences between men and women’s perceptions, with the majority reporting feeling bored (54% men and 43% women). In addition, women (31%) expressed feeling anxious or stressed versus men (24%); others said they felt calm and happy to be at home (13.1% men and 21.4% women). Finally, men (9.3%) expressed feeling more loneliness, discomfort, and frustration than women (5.2%) (see Table 6). These results show that women take confinement better than men, who perceive confinement more negatively.

In terms of general health in pre-existing conditions, the majority reported remaining stable (64% male and 56% female); we could attribute these results to the age of respondents, with 86.4% ranging from 17 to 24 years and 11.5% between 25 and 29 years versus only 2.1% between 30 and 37 years, i.e., youth played a crucial role in good health, as well as keeping busy with classes and university work during the pandemic. Despite this, conditions worsened more in women (19%) than in men (16%). There were no differences between men and women regarding the appearance of new ailments. The main ailments reported by respondents were those related to depression and anxiety (18%), due to the critical situation and uncertainty they had to live, followed by obesity, respiratory problems (16%), and high cholesterol (5%). Finally, rheumatic diseases, circulatory system diseases, diabetes, neurological problems, cancer, and hypertension account for less than 6% (Table 7).

### 3.4. Personal Free Question

**Figure 10:**
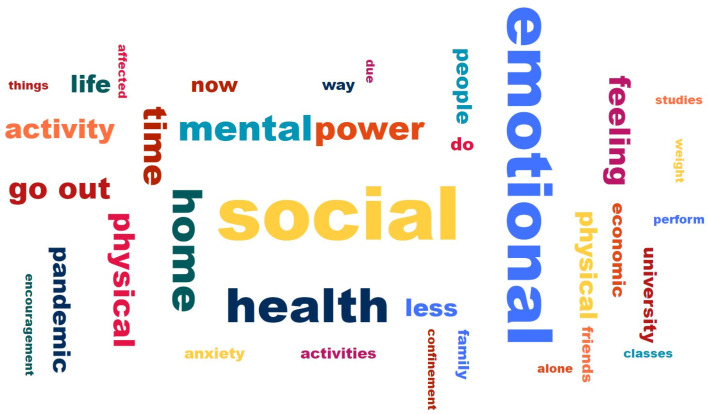
Word cloud of affect factors in college students.

When asked, “In what aspect of your life do you feel that the confinement due to COVID-19 has affected you the most?”, the 375 students (see their characteristics in Table S4 in the Supplementary Material) gave various answers, where the most common words as per the word cloud were as follows: emotional, social, and health (Figure 10). The answers were classified into the following three themes: affectations related to physical activity, affectations related to the emotional aspect, and affectations regarding health during confinement.

#### 3.4.1. Effects Related to Physical Activity during Confinement

The students expressed their discomfort at being unable to carry out physical activities that they used to do, and their discomfort at being unable to frequent places they used to go.

*“Before, I used to do more physical activity when I got to and from the university. I also went to the park to jog and exercise, but during confinement, I have only been able to do a little exercise at home and in a discontinuous way”.*

*(ID 63)*

*“I think I have stopped moving much more than before, and if you add to that sitting for a long time receiving classes, I feel that my physical exercise no longer has the effect I want”.*

*(ID 33)*

*“I have not been able to play the sports I played before (basketball and tennis); because of this, my weight has increased a little and also because, at home, I have a better diet than I had at the university”.*

*(ID 108)*

In addition, specific answers revealed the relationship between physical activity and the economic aspect, as follows:

*“Before the pandemic, I played volleyball, but it has become complicated since they are not open most days, and economically because there are more expenses, which implies less food and so on”.*

*(ID 47)*

#### 3.4.2. Effects Related to the Emotional Aspect

The students reported feeling anxiety, depression, frustration, and stress. It was possible to see that these emotions arose for various reasons, such as being locked up at home, not being able to go out with friends, receiving bad news every day, acquired illnesses, and family losses.

*“Emotionally, I feel overwhelmed at home, unable to go out, being in front of my computer for a long time. Some responsibilities need to do, but not going out and doing them in this space is not so exciting; receiving news and complications at home makes me feel stressed. Socially, it used to be easier to express me in person, and now I feel anxious about seeing crowds gathered together as if I want to be alone instead of being with people around me”.*

*(ID 30)*

*“My mood has worsened. I haven’t spent time with my friends or made any new professional or personal social relationships”.*

*(ID 10)*

*“In the emotional aspect due to the losses that occurred and the seriousness of the situation”.*

*(ID 159)*

Some manifested affectation in the psychological and social aspect.

*“Psychological aspects increased my problems of insecurity and self-esteem”.*

*(ID 176)*

*“In my social life and communication with people, in my state of mind, that is now, I always feel unmotivated, and nothing surprises me. On the contrary, I have become more angry, impatient and bitter. I feel frustrated because I could not meet my goals at a particular stage of my life that I cannot. After all, that time and age have passed”.*

*(ID 328)*

#### 3.4.3. Health Effects during Confinement

The informants reported health problems, such as weight gain, body aches, vision problems, injuries, and mental fatigue, which was exposed by some comments, as follows:

*“In my physical state I have gained weight and adapting has been very complicated for me”.*

*(ID 142)*

*“Carpal tunnel, ear, neck and back pain”.*

*(ID 195)*

*“I am having vision problems, tiredness, migraine, resistance and sometimes I imagine things out of nowhere or talk to myself”.*

*(ID 202)*

*“Injury to my arm and knees”.*

*(ID 148)*

*“Not being able to do physical activity as I did before, I spend more time sitting down, which is detrimental to my health, and spending too much time glued to a computer has caused myopia to increase and mental fatigue to be greater than in person”.*

*(ID 99)*

### 3.5. Teacher Interviews

The teachers who answered the interview were those who taught Dance, Strength Training, Diving and Nautical Activities, as well as professionals in Functional Training. The professionals indicated they had 16 to 29 years of working at ESPOL. They recommend the following physical activities to the students: swimming, soccer, weights, aerobic exercise, such as walking, jogging, or cycling, and focused exercises. For students who did not have an affinity for sports, they recommend frequent walking or mixing a sport with a physical activity that works on body flexibility, such as dance, yoga, or dance therapy.

In addition, they recommend at least 3 to 4 h of physical activity per week. The professional mentions that *“physical activity counteracts the damage the body suffers from prolonged sitting, making the blood flow better, contributing to the assimilation of knowledge during academic activities”*.

The challenges the teachers had during the course were as follows: not having control of each activity and dealing with students who arrived sleepy or with low energy from their previous class. Additionally, they struggled to capture the students’ interest since, due to various problems, they were missing classes.

They recommended recreational and creative activities, such as walking, skating, climbing, and cycling to combat sedentary lifestyles. They also advised students to distribute their daily time between the task of studying, household chores, intra-domiciliary exercise, and relaxation by reading or watching a movie. Finally, they advised students to take planned active breaks during class time.

## 4. Discussion

This study confirmed that university students’ physical activity, emotional, and health status had been affected during the confinement caused by the global pandemic of COVID-19.

Concerning physical activity, it was determined that most of the informants (66.7%) had changed their lifestyle toward a sedentary one. This is because their academic activities, such as receiving classes, studying, and doing homework or projects occupied more than six hours a day. Therefore, there was a substantial reduction in physical activity at this time. These academic activities, together with the severe sanctions for violating confinement [62], limited their ability to participate in organized sports activities [63]. Additionally, the changing characteristics of the neighborhood in which the students live and their environment [64] could be considered the cause of the sedentary behaviour. This reduction in physical activity is presented in other research with Mexican and Italian university students [65]. However, a Spanish study shows the opposite effect, with physical activity increasing by 75% despite confinement. These contrasting results were because people who reported engaging in physical activity, including sports, such as soccer, basketball, and tennis before the pandemic experienced a substantial reduction in activity, while routine activities, such as homework at home increased considerably. The simultaneous combination of restrictions and recommendations to exercise at home during confinement could also influence those who did the physical activity and those who did not [66].

The emotional factor was considered in this study due to the growing concern about mental health effects due to isolation, worries, and fears of social distancing [67]. The analyses determined that anxiety (as a symptom of psychological distress) and stress levels were present in 27.47% of the informants. At the same time, 48% declared feeling bored (Table 4). The students also reported changes in their lifestyle habits, such as sleeping and eating less and even less frequent weekly exercise (Figure 6). This is caused by the interruption of their academic activities (face-to-face, business, or laboratory practices), adaptation to new ways of learning and acquiring knowledge, not having access to sports equipment, gyms, or fitness classes, and the management of the government authorities during the pandemic. As a result, records of depression, anxiety, and anguish during the pandemic are greater than those recorded before the pandemic [68] and are currently considered a global phenomenon [69]. More precisely, in our study, women show higher levels of anxiety or stress, while men show higher levels of boredom.

These emotional problems can also be related to physical activity that requires maintaining a certain level of mobility or being active outdoors. This was reflected by the influence of the community exerting certain psychological pressure due to the fear of being infected by COVID-19, and by policies restricting mobility in access to food, work, and health [70].

The pandemic has caused changes in lifestyles and habits, a reduction in physical activity, and an increase in depression and stress. These changes have caused the informants’ health status to be affected. Obesity and respiratory diseases were the most common, while high cholesterol, rheumatic diseases, diseases affecting the circulatory system, and hypertension were the least common (see Figure 7 and Figure 8). Results are consistent with other studies that mention the population increase in these diseases since the appearance of the pandemic [71,72,73]. Even combinations of these diseases were accentuated, such as in overweight patients with rheumatism [74], arterial diseases with vascular pathologies [75], or the loss of physical and psychological adaptations of athletes due to lack of training caused by confinement [76].

These results follow the trend of similar studies conducted in Latin American [77,78,79,80,81,82] and Caribbean countries [83,84], Spain [47], Italy [85], the United States [86], France, and Switzerland [87], as well as other studies carried out in the United Kingdom, Hong Kong, and China [88].

The university can play a fundamental role in the physical recovery, emotional stability, and health of its students, in the following ways:

(a) Establishment of community development programs, where the participation of the university community is crucial for development [89,90], and its results allow the generation of publications in journals [91,92,93];
(b) Implementing programs that allow organized physical activity and, thus, leads to the reduction in anxiety, depression, and stress, as well as irritability and restlessness [94];
(c) Programs that allow the teaching and learning of adequate nutrition, which would imply a lower intake of foods with a high caloric value or dietary restrictions;
(d) Student mobility programs that allow moving around the university facilities on foot, by bicycle, or on electric scooters, causing an increase in active commuting [64];
(e) The promotion through social networks of activities related to health care, sports activity, and emotional care.

These programs can act as multidimensional strategies, allowing recovery in one or more of the effects studied, resulting in a better sense of tranquility.

The scope of our study was to evaluate the incidence of physical activity on students’ physical and mental health. For this reason, an attempt was made to obtain as much information about their health before and after the pandemic. However, it was impossible to show whether the evolution of pre-existing diseases was due to physical inactivity or the COVID-19 virus, which worsened the health status of many people.

Of the 1321 students sent the survey, 376 responded, and 1 was excluded because they did not complete all the sociodemographic questions. We do not know why those who did not complete the survey chose not to. However, we can speculate a possible response bias of respondents with a particular interest in physical activity.

Another limitation is that the rural or urban area in which they live was not investigated, since this could be an influential factor in the anxiety experienced. It was not asked if the students live with their parents and if their family income is stable, since this influences the people’s mood [95]. However, knowing that most do not work gave us to understand that they depend financially on their parents or a relative. Considering the limitations of this research, it is recommended that future studies include the aforementioned factors.

## 5. Conclusions

This research analyzed the impact of confinement through surveys to students and university professors in order to study its effects on physical activity, emotional state, and health.

In terms of physical activity, 49.6% of students have a low level of physical activity (<300 METs). Most of the respondents, especially women, report 73% sedentary behavior, as they spend more than 6 h a day on academic activities. Emotionally, the data show their perception of the pandemic, with the majority indicating feeling bored. Some expressed anxiety, depression, annoyance, and frustration, while others reported feeling calm and happy at home (see Figure 6).

In terms of health, the affected diseases reported by respondents were rheumatic diseases, circulatory diseases, obesity, high cholesterol, and respiratory diseases (see Figure 7 and Figure 8). In terms of lifestyle habits, those who felt bored increased their sleep and eating (see Figure 8).

Physical activity played an essential role for the students. Those who practiced sport or physical exercise during confinement managed to keep their health stable, although some had pre-existing illnesses. In addition, those who had been physically active faced confinement with a better mood. However, during the pandemic, those who did sport or exercise did not change this habit.

Specifically, according to the results obtained, women have a higher percentage of daily activities compared to men, becoming a conditioning factor that can affect the emotional, health, and physical activity aspects.

The results reinforce the view that sport is a matter of education and culture, which does not change in a pandemic, and requires a discipline of practice. Therefore, there must be a persevering work on exercise and sport, because the habit is a matter of training and education.

At the ESPOL University, as in other universities, there are compulsory subjects of sports and other recreational activities that allow students to relax. However, the performance of these activities does not imply a joint monitoring of their physical, emotional, or health well-being. Therefore, this study would help the student welfare area to correlate the information they have and to intervene in the case of other students who need support.

## Figures and Tables

**Table 1:** Eigenvalues and cumulative variance percentage.

Dimensions	Eigenvalue	Percentage of Variance	Cumulative Percentage of Variance
Dim 1	1.9885	9.0737	9.0737
Dim 2	1.5243	6.9555	16.0292

**Table 2:** Demographic characteristics of respondents.

Variable	Frequency	Percentage (%)
Gender		
Female Male	192 183	51.1 48.9
Age		
16–18 years 18–20 years 20–22 years 22–24 years 24–26 years 26–28 years 28–30 years 32–34 years 36–38 years	16 119 127 63 28 15 5 1 1	4.3 31.7 34.1 16.8 7.5 4.0 1.2 0.2 0.2
Nationality		
Ecuadorian	373	99.4
Venezuelan	1	0.3
Peruvian	1	0.3
Marital status		
Single	366	97.6
Married	6	1.6
Free union	3	0.8
Employment status		
Employed	89	23.7
Unemployed	286	76.3
Faculty		
FADCOM ^1^	104	27.7
FCNM ^2^	98	26.1
FCSH ^3^	52	13.8
FICT ^4^	1	0.3
FCV ^5^	1	0.3
FIEC ^6^	4	1.1
FIMCP ^7^	2	0.5
Not defined	113	30.3

^1^ Faculty of Art, Design, and Audio-visual Communication (FADCOM), ^2^ Faculty of Natural Sciences and Mathematics (FCNM), ^3^ Faculty of Social and Humanistic Sciences (FCSH), ^4^ Faculty of Earth Sciences Engineering (FICT), ^5^ Faculty of Life Sciences (FCV), ^6^ Faculty of Electrical and Computer Engineering (FIEC), ^7^ Faculty of Mechanical Engineering and Production Sciences (FIMCP).

**Table 3:** χ^2^ test (chi square) of stochastic independence.

Categories Evaluated	Result	*p*-Value
Gender vs. PA before the pandemic	Not independent	<0.01
Gender vs. PA during the pandemic	Not independent	<0.01
Gender vs. lifestyle	Not independent	<0.01
Gender vs. emotional state	Not independent	<0.01
Faculty vs. PA before the pandemic	Not independent	<0.01
University vs. PA during the pandemic	Not independent	<0.01
Habits vs. PA during the pandemic	Not independent	<0.01
Emotional state vs. PA during the pandemic	Not independent	<0.01
Illnesses during the pandemic vs. emotional state	Not independent	<0.01

**Table 4:** Physical activity levels and sedentary lifestyles in university students.

Variable		Frequency (Percentage %)		
Activity Level	Avg. MET *	All	Women	Men
Low Moderate High Total	68 (0–300) 794 (300–1500) 1845 (>1500)	186 (49.6%) 155 (41.3%) 34 (9.1%) 375(100%)	106 (55.2%) 72 (37.5%) 14 (7.3%)	80 (43.7%) 83 (45.4%) 20 (10.9%)
Behaviour		All	Women	Men
Sedentary Not sedentary	>6 h sitting <6 h sitting	250 (66.7%) 125 (33.3%)	140 (72.9%) 52 (27.1%)	110 (60.1%) 73 (39.9%)
Total		375 (100%)		

* Here, Avg. MET—average MET.

**Table 5:** Physical activity before and during the pandemic.

Physical Activities	Men		Women		Total	
	Before	During	Before	During	Before	During
Daily activities (climbing stairs, doing housework)	11%	20%	27%	36%	19%	29%
Sports (soccer, basketball, tennis, etc.)	45%	15%	18%	5%	31%	10%
Aerobic exercise (jogging, walking, cycling)	26%	23%	34%	28%	30%	26%
Coordination exercise (dancing)	0%	1%	8%	10%	4%	6%
None of the above	8%	22%	7%	16%	8%	19%
Passive recreation (board game, playing an instrument, attending language academies)	3%	5%	2%	2%	2%	3%
Muscular endurance (lifting loads).	8%	14%	5%	3%	6%	8%
Total	100%	100%	100%	100%	100%	100%

**Table 6:** Emotional state and health status.

Emotional State	Men	Women	Total
Boredom	53.6%	42.7%	48.0%
Anxiety or stress	24.0%	30.7%	27.5%
Tranquility	10.4%	15.6%	13.1%
Happy to be home	2.7%	5.7%	4.3%
Loneliness	4.9%	2.6%	3.7%
Frustration and annoyance	4.4%	2.6%	3.5%
Total	100%	100%	100%
Health status			
My illness has worsened	16%	19%	17%
My health has improved	10%	13%	11%
New diseases have appeared	10%	12%	11%
My health remains stable before and during confinement	64%	56%	60%
Total	100%	100%	100%

**Table 7:** Perception of pre-existing diseases during confinement.

Pre-Existing Diseases	My Illness Has Worsened	My Health Has Improved	New Diseases Have Appeared	My Health Remains Stable before and during Confinement	Total
None	2%	6%	7%	85%	45%
Depression and anxiety	30%	20%	11%	39%	18%
Other	17%	10%	24%	50%	11%
Obesity	49%	5%	13%	33%	10%
Respiratory problems	14%	19%	14%	52%	6.0%
High cholesterol	35%	29%	12%	24%	5.0%
Rheumatic diseases	40%	20%	40%	0%	1.3%
Circulation problems	60%	0%	20%	20%	1.3%
Diabetes	0%	67%	0%	33%	0.8%
Neurological problems	33%	33%	0%	33%	0.8%
Cancer	0%	0%	0%	100%	0.5%
Hypertension	50%	50%	0%	0%	0.5%

## Data Availability

Not applicable.

## Supplementary Materials

The following are available online at https://www.mdpi.com/article/10.3390/ijerph191711016/s1, Figure S1. Type of physical activity, Table S1: Sample estimation formulas, Table S2: Survey of university students, Table S3: Questions for teacher’s interviews, Table S4: Participant characteristics.
