# Supplementary material for: Multivariate Analysis on Physical Activity, Emotional and Health Status of University Students Caused by COVID-19 Confinement
Source: Int J Environ Res Public Health. 2022 Sep 3;19(17):11016. doi: 10.3390/ijerph191711016 (PMC9518169; doi:10.3390/ijerph191711016)
Supplement: Supplementary file 1 [file ijerph-19-11016-s001.zip › ijerph-1843779-supplementary.pdf]

Supplementary Table S1: Sample estimation formulas

Initial sample size n_o

This equation 1, for a population greater than 10,000:

$$n_o = \frac{Z^2 * p * q}{e^2} \tag{1}$$

n_o = Initial sample size for a simple random sample.
Z = Statistical parameter that depends on the confidence level (percentile of a standard normal distribution).
p = The probability of occurrence of the studied event (success).
$q = (1 - p)$ = The probability that the event studied will not occur.
e = Maximum accepted estimation error

Population adjustment n'

$$n' = \frac{n_o}{1 + \frac{(n_o - 1)}{N}} \tag{2}$$

n' = Adjusted population

N = Size of the population or universe of the population

Supplementary Table S2: Survey of university students

The Escuela Superior Politécnica del Litoral ESPOL of Ecuador is conducting a study on the "Effects of physical and emotional activity and health status due to COVID-19 confinement in university students". Your authorization is requested to participate in this research project whose objective is to analyze the impact of confinement due to the pandemic. Your participation is completely voluntary, if you do not wish to participate, there will be no negative consequences. You may withdraw from the study at any time. The response is completely anonymous. If you have any questions, please contact Mayra Brocel/Bethy Merchán, whose e-mail addresses are: mbrocel@espol.edu.ec and betgumer@espol.edu.ec.

I do agree to participate ❑ I do not agree to participate ❑

Sociodemographic aspects

**No.**	**Question**	**Response**
1.	Gender	Male<br>Feminine
2.	Age	
3.	Marital status	Single<br>Married<br>Divorced<br>Widower<br>Free union
4.	Nationality	
5.	No. registration	
6.	Indicate your faculty and career	

7.	Are you currently working? (Q1)	Yes<br>No
**Health aspects**		
8.	Which of these diseases do you have or have you had? (Q2)	Obesity<br>Hypertension<br>Diabetes<br>High cholesterol<br>Cancer<br>Circulation problems<br>Rheumatic diseases<br>Respiratory problems<br>Neurological problems<br>Depression and anxiety<br>Other ()<br>None ()
9.	During confinement, how have the aforementioned diseases changed? (Q3)	My illness has worsened.<br>My health has improved.<br>New diseases have appeared.<br>My health remains stable before and during confinement.
10.	How have your life habits changed, due to confinement? (Q4)	I sleep less than before.<br>I sleep more than before.<br>My hours of sleep remain the same before and during the pandemic.<br>I do less weekly physical exercise.<br>I do more weekly physical exercise.<br>My physical activity remains the same before and during the pandemic.<br>I eat more than before.<br>I eat less than before.<br>I eat the same before and during the pandemic.
**Aspects of moods**		
11.	What emotions best represent your mood, being at home? (Q5)	Boredom.<br>Anxiety or stress.<br>Frustration and annoyance.<br>Loneliness.<br>Tranquillity.<br>Happy to be home.
**Sports aspects**		
To answer the following questions, remember that: VIGO-ROSAS physical activities are those that require great physical effort and that make you breathe MUCH harder than normal. MODERATE physical activities are those that require some physical effort and make you breathe a LITTLE harder than normal.		

12.	Select the types of physical activity you did weekly BEFORE the pandemic (Q6)	Daily activities (climbing stairs, doing housework).<br>Sports (soccer, basketball, tennis, etc).<br>Aerobic exercise (jogging, walking, cycling).<br>Coordination exercise (dancing).<br>Passive recreation (board game, playing an instrument, attending language academies).<br>Muscular endurance (lifting loads).<br>None of the above.
13.	Select the types of physical activity you do weekly DURING the pandemic(Q7)	Daily activities (climbing stairs, doing housework).<br>Sports (soccer, basketball, tennis, etc).<br>Aerobic exercise (jogging, walking, cycling).<br>Coordination exercise (dancing).<br>Passive recreation (board games, playing an instrument, attending language academies).<br>Muscular endurance (lifting loads). None of the above.
14.	How many days/week do you do VIGOROUS physical activity? (Q8)	2 to 3<br>4 to 5<br>More than 5<br>None
15.	How many minutes/days do you do VIGOROUS physical activity? (Q9)	10 to 30 minutes<br>30 to 40 minutes<br>More than 40 minutes<br>Less than 10 minutes
16.	How many days/week do you do MODERATE physical activity? (Q10)	2 to 3<br>4 to 5<br>More than 5<br>None
17.	How many minutes/days do you do MODERATE physical activity? (Q11)	10 to 30 minutes<br>30 to 40 minutes<br>More than 40 minutes<br>Less than 10 minutes
18.	How many hours do you spend sitting down receiving classes, studying, or doing projects or homework? (Q12)	3 hours or less<br>4 to 6 hours<br>6 to 8 hours<br>More than 8 hours
**Personal question**		
19.	In what aspect of your life do you feel that the confinement due to COVID-19 has affected you the most? (Q13)	

Supplementary Table S3: Questions for teacher´s interviews

1. ¿What complementary subject do you teach at ESPOL?
2. ¿What physical activities do you recommend ESPOL students do to improve their quality of life?
3. ¿How often (hours/week) do you recommend doing physical activity to polytechnic students (adults)?
4. ¿How many years have you been teaching classes at ESPOL (indicate the duration and start year)?
5. What challenges have you had teaching classes in this time of pandemic?
6. Recommend some other way to help combat the sedentary lifestyle in polytechnic students.

Supplementary Table S4: Participant characteristics

ID	Gender	Age	Marital status	Employment status
1	Male	24	Single	Yes
2	Female	18	Single	No
3	Female	17	Single	No
4	Female	20	Single	No
5	Female	23	Single	Yes
6	Male	27	Single	No
7	Male	19	Single	No
8	Female	18	Single	No
9	Male	20	Single	Yes
10	Female	20	Single	Yes
11	Male	21	Single	No
12	Male	19	Single	No
13	Male	21	Single	No
14	Female	18	Single	No
15	Male	20	Single	No
16	Male	24	Single	Yes
17	Female	25	Single	No
18	Male	19	Single	No
19	Female	25	Single	No
20	Female	22	Single	No
21	Female	25	Single	No
22	Male	22	Single	No
23	Male	23	Single	No
24	Female	22	Single	No
25	Female	22	Single	No
26	Female	22	Single	Yes
27	Female	20	Single	Yes
28	Female	18	Single	Yes
29	Female	21	Single	No
30	Female	20	Single	No
31	Female	27	Single	No
32	Female	22	Single	No

33	Female	19	Single	No
34	Female	28	Single	No
35	Female	22	Single	No
36	Female	20	Single	No
37	Female	21	Single	No
38	Male	20	Single	No
39	Female	19	Single	No
40	Male	24	Single	No
41	Male	21	Single	Yes
42	Female	20	Single	No
43	Female	19	Single	No
44	Female	23	Single	Yes
45	Female	24	Single	No
46	Female	21	Single	No
47	Female	20	Single	No
48	Male	21	Single	No
49	Male	19	Single	Yes
50	Male	21	Single	No
51	Male	22	Single	Yes
52	Female	21	Single	No
53	Male	20	Single	No
54	Female	21	Single	No
55	Male	24	Single	No
56	Female	20	Single	Yes
57	Female	19	Single	No
58	Female	21	Single	No
59	Female	25	Single	No
60	Male	24	Single	Yes
61	Female	24	Single	No
62	Female	20	Single	No
63	Female	22	Single	No
64	Male	19	Single	No
65	Female	24	Single	No
66	Male	20	Single	No
67	Female	21	Single	No
68	Female	18	Single	No
69	Female	20	Single	No
70	Male	22	Single	No
71	Male	22	Single	No
72	Male	18	Single	No
73	Male	21	Single	No
74	Male	23	Single	No
75	Male	20	Single	No
76	Female	20	Single	No
77	Female	18	Single	No
78	Male	21	Single	Yes
79	Male	26	Single	Yes
80	Male	19	Single	No
81	Male	18	Single	No
82	Male	20	Single	No
83	Male	27	Single	No

84	Male	23	Single	No
85	Male	20	Single	No
86	Female	18	Single	No
87	Male	24	Single	Yes
88	Male	21	Single	No
89	Female	20	Single	No
90	Male	21	Single	No
91	Female	24	Single	No
92	Male	20	Single	Yes
93	Male	20	Single	Yes
94	Female	20	Single	Yes
95	Female	24	Single	Yes
96	Male	18	Single	No
97	Female	20	Single	No
98	Male	21	Single	No
99	Male	20	Single	No
100	Female	20	Single	No
101	Male	27	Single	No
102	Female	19	Single	No
103	Female	27	Married	Yes
104	Male	19	Single	No
105	Male	19	Single	No
106	Female	18	Single	No
107	Female	21	Single	No
108	Female	23	Single	No
109	Male	21	Single	Yes
110	Male	20	Single	No
111	Female	20	Single	No
112	Male	19	Single	No
113	Female	24	Single	No
114	Male	19	Single	No
115	Male	24	Single	Yes
116	Female	20	Single	No
117	Male	22	Single	No
118	Male	20	Single	No
119	Female	21	Single	No
120	Female	22	Single	No
121	Male	20	Single	No
122	Male	20	Single	No
123	Female	28	Single	No
124	Female	19	Single	No
125	Female	19	Single	No
126	Female	19	Single	No
127	Female	19	Single	No
128	Male	25	Single	No
129	Male	26	Single	Yes
130	Female	22	Single	No
131	Female	20	Single	Yes
132	Female	22	Single	Yes
133	Male	22	Single	No
134	Female	22	Single	No

135	Male	25	Single	No
136	Male	25	Single	No
137	Male	26	Single	No
138	Female	18	Single	No
139	Male	20	Single	No
140	Female	20	Free union	No
141	Male	22	Single	No
142	Female	19	Single	No
143	Male	29	Single	No
144	Female	20	Single	No
145	Male	21	Single	No
146	Female	19	Single	No
147	Female	24	Single	Yes
148	Male	23	Single	No
149	Male	20	Single	Yes
150	Male	20	Single	Yes
151	Female	20	Single	No
152	Female	22	Single	No
153	Female	20	Single	Yes
154	Female	21	Single	Yes
155	Male	21	Single	No
156	Female	21	Single	Yes
157	Female	Q9	Single	No
158	Male	21	Single	No
159	Female	22	Single	No
160	Female	20	Single	No
161	Female	23	Single	No
162	Female	21	Single	Yes
163	Male	21	Single	No
164	Male	21	Single	No
165	Female	20	Single	Yes
166	Male	20	Single	No
167	Female	21	Single	No
168	Female	20	Single	No
169	Male	21	Single	No
170	Male	21	Single	No
171	Female	19	Single	No
172	Female	20	Single	No
173	Female	20	Single	No
174	Male	23	Single	No
175	Female	19	Single	No
176	Female	20	Single	No
177	Male	20	Single	No
178	Male	21	Single	Yes
179	Female	23	Free union	Yes
180	Female	25	Single	No
181	Female	21	Single	No
182	Female	20	Single	No
183	Male	25	Single	No
184	Male	19	Single	No
185	Female	21	Single	

186	Female	22	Single	No
187	Male	22	Single	No
188	Male	22	Single	No
189	Female	21	Single	No
190	Female	22	Single	No
191	Female	26	Single	No
192	Female	21	Single	Yes
193	Female	18	Single	No
194	Female	22	Single	Yes
195	Female	28	Single	Yes
196	Female	19	Single	No
197	Female	21	Single	No
198	Male	24	Single	No
199	Male	21	Single	No
200	Male	22	Single	Yes
201	Female	19	Single	No
202	Male	21	Single	Yes
203	Female	24	Single	No
204	Male	20	Single	No
205	Male	25	Single	No
206	Male	20	Single	Yes
207	Male	19	Single	No
208	Male	25	Single	No
209	Female	23	Single	Yes
210	Male	25	Single	No
211	Male	20	Single	No
212	Male	27	Single	Yes
213	Male	21	Single	Yes
214	Male	22	Single	No
215	Female	21	Single	Yes
216	Male	22	Single	No
217	Male	21	Single	Yes
218	Female	27	Single	Yes
219	Male	24	Single	No
220	Male	21	Single	No
221	Male	23	Single	Yes
222	Female	20	Single	No
223	Female	20	Single	No
224	Female	25	Single	No
225	Male	21	Single	Yes
226	Female	21	Single	No
227	Male	20	Single	No
228	Male	20	Single	No
229	Male	21	Single	No
230	Female	25	Single	No
231	Male	19	Single	Yes
232	Male	21	Single	No
233	Male	21	Single	No
234	Female	20	Single	No
235	Male	29	Single	Yes
236	Female	20	Single	No

237	Male	21	Single	No
238	Male	19	Single	No
239	Male	19	Single	No
240	Female	24	Single	Yes
241	Male	20	Single	Yes
242	Male	23	Single	Yes
243	Male	20	Single	No
244	Male	24	Single	No
245	Male	20	Single	No
246	Male	21	Single	Yes
247	Male	24	Single	No
248	Female	19	Single	Yes
249	Female	22	Single	No
250	Male	21	Single	Yes
251	Female	20	Single	No
252	Female	21	Single	Yes
253	Male	22	Single	No
254	Female	34	Single	No
255	Male	22	Single	Yes
256	Female	23	Single	No
257	Male	20	Single	No
258	Female	22	Single	Yes
259	Male	23	Single	No
260	Female	21	Single	No
261	Female	22	Single	No
262	Male	24	Single	No
263	Female	19	Single	No
264	Male	21	Single	Yes
265	Male	24	Single	No
266	Male	22	Single	No
267	Female	23	Married	No
268	Male	21	Single	No
269	Female	25	Single	Yes
270	Female	20	Single	No
271	Female	24	Single	No
272	Female	27	Married	No
273	Female	20	Single	No
274	Male	25	Single	No
275	Female	23	Single	No
276	Female	20	Single	No
277	Male	21	Single	No
278	Female	27	Single	Yes
279	Female	20	Single	No
280	Male	22	Single	No
281	Female	25	Single	No
282	Female	24	Single	No
283	Female	20	Single	No
284	Female	24	Single	No
285	Female	23	Single	Yes
286	Female	23	Married	No
287	Male	25	Single	No

288	Male	21	Single	No
289	Male	25	Single	Yes
290	Male	28	Single	No
291	Male	20	Single	No
292	Female	24	Single	No
293	Male	23	Single	No
294	Male	21	Single	No
295	Female	22	Single	No
296	Female	21	Single	No
297	Male	27	Single	Yes
298	Female	26	Single	No
299	Female	22	Single	No
300	Male	23	Single	No
301	Female	21	Single	No
302	Male	20	Single	No
303	Female	21	Single	No
304	Male	25	Single	No
305	Female	20	Single	No
306	Female	29	Single	No
307	Female	20	Single	No
308	Female	19	Single	No
309	Female	22	Single	No
310	Male	22	Single	No
311	Male	23	Single	No
312	Male	22	Single	Yes
313	Female	21	Single	No
314	Male	22	Single	No
315	Male	22	Single	Yes
316	Male	18	Single	No
317	Male	21	Single	Yes
318	Male	23	Single	Yes
319	Female	23	Single	No
320	Male	30	Single	No
321	Female	29	Married	No
322	Female	22	Single	No
323	Male	21	Single	Yes
324	Male	23	Single	No
325	Female	23	Single	Yes
326	Male	23	Single	Yes
327	Male	23	Single	No
328	Female	22	Single	Yes
329	Male	19	Single	Yes
330	Male	21	Single	No
331	Female	23	Single	No
332	Male	23	Single	No
333	Female	21	Single	No
334	Female	20	Single	No
335	Female	24	Single	No
336	Male	21	Single	Yes
337	Male	21	Single	No
338	Male	20	Single	No

339	Male	22	Single	Yes
340	Male	18	Single	No
341	Male	21	Single	No
342	Male	20	Single	No
343	Female	23	Single	Yes
344	Male	23	Single	No
345	Male	19	Single	No
346	Female	21	Single	No
347	Male	37	Married	Yes
348	Female	21	Single	No
349	Male	20	Single	No
350	Male	21	Single	Yes
351	Female	25	Single	No
352	Male	19	Single	Yes
353	Female	20	Single	No
354	Female	21	Single	No
355	Male	21	Single	No
356	Female	21	Single	No
357	Male	21	Single	Yes
358	Male	22	Single	No
359	Female	20	Single	No
360	Male	21	Free union	Yes
361	Female	20	Single	No
362	Female	20	Single	Yes
363	Female	21	Single	No
364	Male	22	Single	No
365	Female	21	Single	No
366	Female	24	Single	Yes
367	Male	22	Single	No
368	Female	22	Single	No
369	Female	23	Single	Yes
370	Female	25	Single	No
371	Male	20	Single	No
372	Female	19	Single	No
373	Female	27	Single	No
374	Female	24	Single	No
375	Male	25	Single	Yes

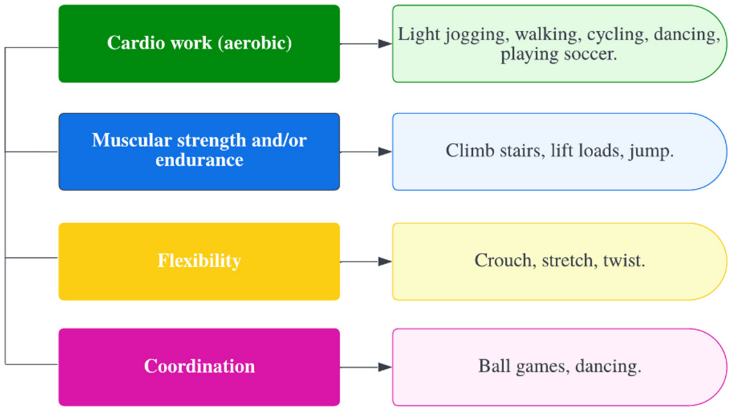

Supplementary Figure S1. Type of physical activity [12].
